# *Trichinella spiralis* Excretory-Secretory Products Protect against Polymicrobial Sepsis by Suppressing MyD88 via Mannose Receptor

**DOI:** 10.1155/2014/898646

**Published:** 2014-06-26

**Authors:** Linlin Du, Lihua Liu, Yang Yu, Hui Shan, Leiqing Li

**Affiliations:** ^1^Department of Intensive Care Unit, The Second Affiliated Hospital of Zhejiang University School of Medicine, 88 Jiefang Road, Hangzhou 310009, China; ^2^Department of Respiratory Disease, The First Affiliated Hospital of Guangxi Medical University, Nanning 530000, China

## Abstract

*Trichinella spiralis* (*T. spiralis*) or its excretory-secretory products (TsES) protect hosts from autoimmune diseases, which depend on inducing host T helper (Th) 2 immune response and inhibiting inflammatory factors. Sepsis is a systemic inflammatory response syndrome (SIRS) evoked by infection. Little is known about the effects of helminths or their excretory-secretory products on sepsis. Here, we investigated the effects of TsES in a mice model of polymicrobial sepsis. TsES improved survival, reduced organ injury, and enhanced bacterial clearance in septic mice. To investigate the molecular mechanism, macrophages from septic patients or the control group were incubated with TsES. TsES reduced sepsis-inducing inflammatory cytokines mediated by Toll-like receptors (TLR) *in vitro* by suppressing TLR adaptor-transducer myeloid differentiation factor 88 (MyD88) and nuclear factor- (NF-)-*κ*B. Furthermore, TsES upregulated mannose receptor (MR) expression during sepsis. MR blocking attenuated the effects of TsES on MyD88 and NF-*κ*B expression. *In vivo*, MR RNAi reduced the survival rate of septic mice treated with TsES, suggesting that TsES-mediated protection against polymicrobial sepsis is dependent on MR. Thus, TsES administration might be a potential therapeutic strategy for treating sepsis.

## 1. Introduction

Sepsis is defined as the systemic inflammatory response syndrome (SIRS) that occurs during an infection. Sepsis is a huge and expensive medical problem worldwide and also is the most common cause of mortality in the intensive care unit (ICU) [1]. Bacteria products, such as lipoteichoic acid and bacterial lipopolysaccharide (LPS), can trigger SIRS and lead to the production of inflammatory mediators during sepsis. Toll-like receptors (TLR) signaling like TLR4 and TLR2 is the key pathway in sepsis pathophysiology [2]. Apart from extracellular TLR part in the signaling cascade, there are also such “participants” as a cytoplasmic part referred to as adaptor protein myeloid differentiation factor 88 (MyD88), TNF-a receptor-associated factor 6 (TRAF6), IL-1 receptor-associated kinase (IRAK), nuclear factor (NF)-*κ*B, and so on. This signaling process results in the production of cytokines including tumor necrosis factor (TNF)-*α*, interleukin- (IL-) 1*β*, IL-6, and IL-10, transforming growth factor (TGF) *β*1, and the late mediator high mobility group box-1 protein (HMGB1) [3]. The aberrant production of proinflammatory mediators can lead to multiorgan failure and even death. Although numerous signaling cascades are triggered during septic shock, clinical trials with biomodulators to block or inhibit inflammatory signaling pathways have generally failed to improve outcomes in patients with sepsis [4].

*Trichinella spiralis* (*T. spiralis*) is one of the most widespread zoonotic parasitic nematodes in the world. Interestingly, infection with* T. spiralis* could protect hosts from autoimmune diseases such as inflammatory bowel disease (IBD) [5], type I diabetes mellitus [6], and autoimmune encephalomyelitis [7]. This effect is not limited to* T. spiralis, *but also includes* Schistosome eggs, T. muris, or Hymenolepis diminuta *which have been shown to protect rats or mice from autoimmune diseases like IBD and asthma [8]. The mechanism is that helminth or their antigens or excretory-secretory antigens have been shown to induce host T helper (Th) 2 immune response and produce anti-inflammatory factors (IL-4, IL-5, and IL-13) and regulatory factors (IL-10, TGF*β*1), which could inhibit Th1 inflammatory factors (e.g., TNF-*α*, IL-1*β*, and IL-6) and Th17 immune response [9, 10].* In vitro, T. spiralis* excretory-secretory antigens (TsES) significantly reduce TNF-*α*, IL-1*β*, IL-6, and IL-12 by inhibiting NF-*κ*B and mitogen-activated protein kinase (MAPK) in LPS-stimulated macrophages [11]. TsES also reduce proinflammatory factors in LPS-stimulated dendritic cells (DC) [12]. Therefore, TsES have an immunomodulatory effect on T cells, macrophages, and DC.

TsES have been characterized as cystatins, serpins, glycans, mucins, lectins, or cytokine homologs which could be potential immunomodulators [13]. TsES are rich in oligomannose residues and contain various carbohydrates and glycoproteins that can potential ligands for various C-type lectin receptors (CLR) such as mannose receptor (MR) [14]. The MR is a pattern recognition receptor of the innate immune system expressed on the surface of macrophages, DC, and some epithelial cells, that binds to microbial structures bearing mannose to mediate endocytosis and phagocytosis, as well as activation of macrophages and DC and antigen presentation [15, 16]. Studies have shown that* T. spiralis* can recognize MR and affect IL-6 and NO expression in macrophages [14]. In addition, MR recognition of* T. muris* components produces an effective immune response that leads to macrophage activation [17].

Because of the anti-inflammatory role of TsES and their potential importance as MR ligands, we hypothesized that TsES might control SIRS during polymicrobial sepsis. To investigate this, we established a polymicrobial sepsis model in mice using cecal ligation and puncture (CLP). We found that treatment with TsES improved the survival rate of septic mice. In addition, TsES inhibited the TLR signaling pathway MyD88 and NF-*κ*B and regulated the production of pro- and anti-inflammatory factors via MR* in vitro* and* in vivo*. The results of this study suggest that TsES should be investigated as a novel therapeutic strategy for sepsis.

## 2. Materials and Methods

### 2.1. Preparation of* Trichinella spiralis* Excretory-Secretory Products

Muscle larvae were recovered from the mice infected with* T. spiralis* by artificial digestion. Preparation of ES proteins was performed as previously described [18]. Briefly, after washing in sterile saline and serum-free RPMI-1640 medium, the larvae were incubated in serum-free RPMI-1640 medium at 3,000 worms/mL for 18 h at 37°C. After incubation, the supernatant was collected and filtered through a 0.2 *μ*m membrane. The ES products were dialyzed and then lyophilized by vacuum concentration and freeze-drying (Heto Maxi-Dry-Lyo, Denmark). The protein concentration was determined by the Bradford assay.

### 2.2. Mouse Experiments

Male Balb/c mice aged 6–8 weeks were purchased from the experimental animal center at Zhejiang University. Experiments followed a protocol drafted by the Animal Ethics Committee of the University. A clinically relevant rodent model of sepsis was created by CLP as has been described previously [19]. Briefly, mice were anesthetized with ketamine (100 mg/kg). The abdominal cavity was opened in layers. The cecum was ligated 1.0 cm from the tip. A through-and-through puncture was made with an 18-gauge needle, and a small amount (droplet) of feces was extruded to ensure the patency of the puncture site before returning it back to the abdominal cavity. The control mice underwent a sham-operation receiving a laparotomy but no CLP. The mice were randomly divided into four groups as follows: PBS + sham, PBS + CLP, TsES + CLP, and TsES + sham. Each mouse was intraperitoneally administrated with 50 *μ*g TsES or the same volume PBS at the end of sham or CLP operation. The survival rate, organ function, and white cells were monitored over the following 4 days.

### 2.3. Patients and Peritoneal Macrophage Cell Culture

We prospectively enrolled 43 patients after abdominal surgery in the Intensive Care Unit of the Second Affiliated Hospital of Zhejiang University School of Medicine from August 2012 to September 2013. 27 patients received treatment for sepsis and 16 patients were recruited as nonseptic controls. All patients presented clinical and/or laboratory variables that fulfilled the criteria for sepsis [20]. The study was approved by the Human Ethics Committee of Zhejiang University and written informed consent was obtained from the patients. Macrophages were collected from the peritoneal lavage or abdominal cavity flushing fluid. The recovered cells were washed with PBS three times. Macrophages were finally resuspended in Dulbeco's modified Eagle's medium (DMEM) supplemented with 10% fetal bovine serum (Invitrogen, Carlsbad, CA, USA), 100 U/mL penicillin, and 100 *μ*g/mL streptomycin (Sigma, St. Louis, MO, USA) and allowed to adhere for 2 h at 37°C, 5% CO_2_. Nonadherent cells were removed by washing and the cultured cells contained >92% macrophages. Macrophages were treated with mouse anti-human MR IgG1 (5 *μ*g/mL) or TsES (5 *μ*g/mL) or their combination. Mouse IgG1 was the isotype control (Santa Cruz, CA, USA).

### 2.4. Quantification of Bacterial (CFU)

5 *μ*L of peritoneal lavage fluid or blood from mice at 4 h and 24 h after the CLP operation was diluted in sterile 0.9% NaCl. Each diluted sample (5 *μ*L) was aseptically plated and cultured at 37°C on nutrient agar plates for nonfastidious microorganism plates. After 24 h, the numbers of bacteria colony-forming units (CFU) were counted.

### 2.5. Cytokines ELISA

The serum concentrations of the cytokines TNF-*α*, IL-1*β*, IL-6, HMGB1, IL-10, and TGF*β*1 were measured at 12 h after the CLP operation by enzyme-linked immunosorbent assay (ELISA). Mouse ELISA kits were purchased from R&D Systems (Minneapolis, MN, USA). The culture supernatants from patient peritoneal macrophages were collected and assayed for TNF-*α*, IL-1*β*, IL-10, and TGF*β*1 by ELISA according to the manufacturer's instructions. Human ELISA kits were purchased from eBioscience (San Diego, CA, USA). 

### 2.6. Western Blot

The cytoplasmic and membrane protein fractions from treated human peritoneal macrophages and homogenized mouse liver tissues were isolated using extraction reagents according to the manufacturer's protocol (KeyGEN Biotech, Nanjing, China). The protein concentrations in all of the samples were determined by using the Bradford Protein Assay Kit (KeyGEN Biotech, Nanjing, China). Western blotting was performed as described previously [9]. The following antibodies were purchased from Santa Cruz (CA, USA): mouse anti-human TLR2, mouse anti-human TLR4, mouse anti-human MR, mouse anti-human MyD88, rabbit anti-mouse MyD88, rabbit anti-mouse MR, rabbit anti-mouse *β*-actin, and mouse anti-human *β*-actin. Immunoreactivity was detected using a horseradish peroxidase-conjugated second antibody and an enhanced chemiluminescence reaction (Pierce, Rockford, IL, USA).

### 2.7. NF-*κ*B Activity

NF-*κ*B activity was determined in nuclear extracts using the NF-*κ*B p50/p65 Transcription Factor Assay Kit (Chemicon, Temecula, CA) according to the manufacturer's instructions. Absorbance at 450 nm was analyzed using an automated plate reader (Bio-Rad Laboratories, Hercules, CA).

### 2.8. MR siRNA Transfer* In Vivo*

MR small interfering RNA (siRNA) (sc-45361) and control siRNA (sc-37007) were purchased from Santa Cruz Biotechnology, Inc. (CA, USA). Entranster—*in vivo *transfection reagent was purchased from Engreen Biosystem Co., Ltd. (Beijing, China). The Entranster—*in vivo*—siRNA mixture was prepared according to the manufacturer's instructions. The method performed has been described previously [21]. Briefly, 5 *μ*g of MR siRNA or control siRNA (con siRNA) was dissolved in 5 *μ*L of RNase-free water. Next, 5 *μ*L of MR siRNA or con siRNA solution was mixed with 5 *μ*L of the Entranster—*in vivo *transfection reagent. The mixture was injected via the tail vein into each mouse using a microsyringe 3 and 5 days before the CLP operation. Mice were randomly divided into six groups as follows: (1) CLP, (2) con siRNA + CLP, (3) MR siRNA + CLP, (4) con siRNA + TsES + CLP, (5) MR siRNA + TsES + CLP, and (6) TsES + CLP. All of the mice received either 50 *μ*g TsES or the same volume PBS at the end of the CLP as indicated.

### 2.9. Statistical Analysis

Survival curves were analyzed using the log-rank (mantel-cox) test. The unpaired Student's* t*-test was used to determine the statistical significance between groups. Data are expressed as means ± SD. *P* values below 0.05 were considered significant. All experiments were performed in triplicate and equivalent results were obtained in each experiment.

## 3. Results

### 3.1. TsES Improved Survival from CLP-Induced Sepsis In mice

**Figure 1:**
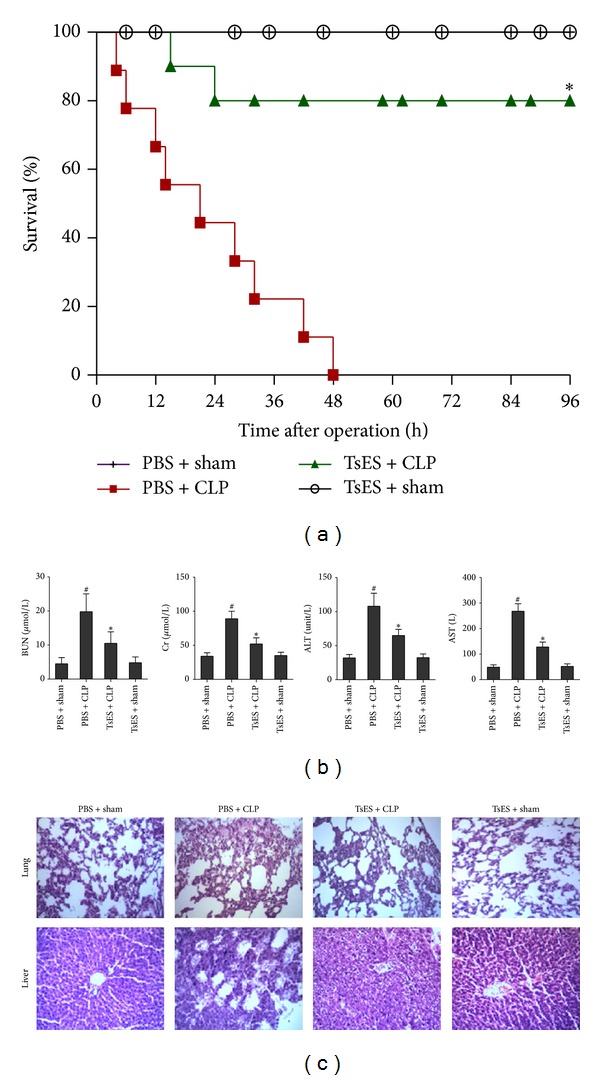
TsES improved survival in a CLP-induced sepsis model. Mice were anesthetized for the CLP or sham operation and treated with 50 *μ*g TsES or the same volume of PBS. Mice were randomly divided into four groups (*n* = 10 mice/group): PBS + sham, PBS + CLP, TsES + CLP, and TsES + sham, and observed for 4 days after the operation. The survival rate was analyzed using the log-rank test. *P < 0.05 compared with the PBS + CLP group (a). Liver and kidney function markers (ALT, AST, BUN, and Cr) from mice in each of the four groups were assessed 24 h after the operation. ^#^ *P* < 0.05 compared with PBS + sham mice. *P < 0.05 compared with PBS + CLP group (b). Lung and liver sections from mice treated with TsES were assessed 24 h after the operation. Scale bars, 50 *μ*m (c).

We employed a CLP-induced murine sepsis model to determine whether administering TsES could protect mice from sepsis. Administration of a single dose (50 *μ*g/mouse) of TsES intraperitoneally reduced mortality and was sufficient to protect the mice from CLP-induced death (Figure 1(a)). The function of the kidney and liver was examined in mice 24 h after the CLP operation. Treatment with TsES attenuated the increased serum levels of ALT, AST, BUN, and Cr caused by CLP-induced sepsis, indicating less organ damage. TsES did not impair organ function (Figure 1(b)). Analysis of tissue samples from the mice treated with TsES showed substantial suppression of CLP-induced pathology in terms of both immune cell infiltrates and damage to the lungs and liver (Figure 1(c)).

### 3.2. TsES Reduced the Inflammatory Response and Enhanced Bacterial Clearance

**Figure 2:**
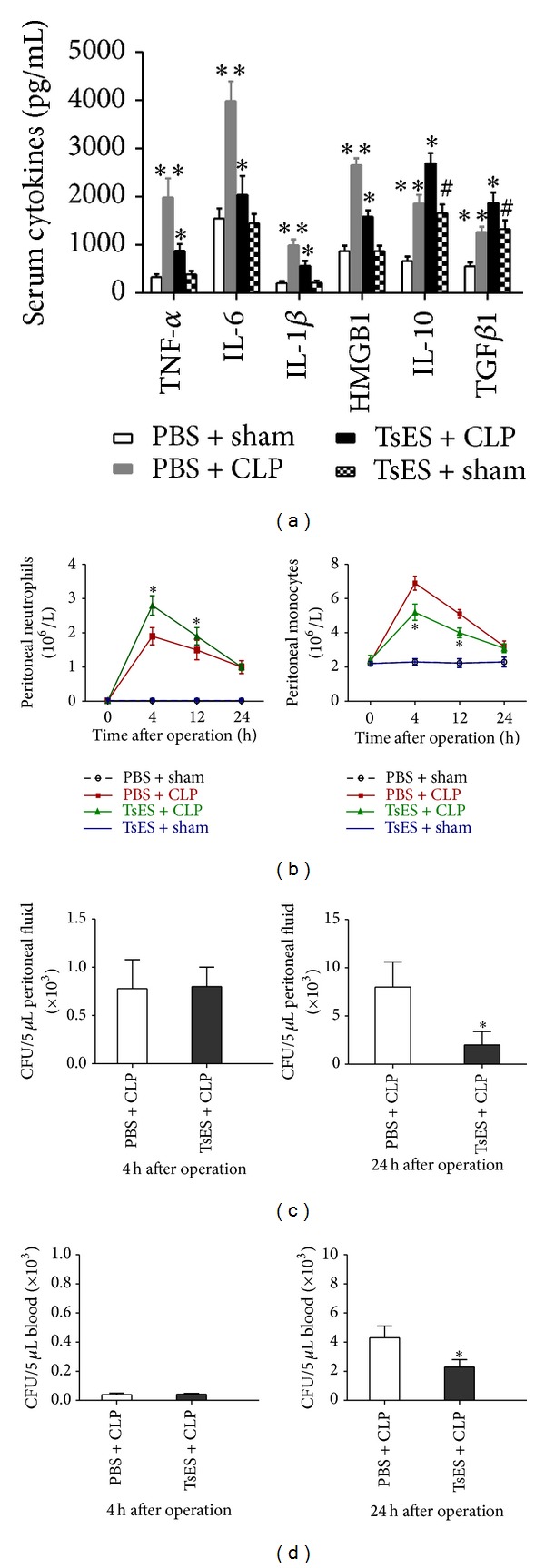
TsES reduced the inflammatory response and enhanced bacterial clearance. Serum TNF-*α*, IL-1*β*, IL-6, HMGB1, IL-10, and TGF*β*1 levels in mice 12 h after the operation were measured by ELISA (a). Mouse peritoneal neutrophils and monocytes were counted 4 h, 12 h, and 24 h after the CLP or sham operation (b). Peritoneal lavage fluid (c) and blood (d) obtained 4 h and 24 h after the CLP operation were cultured on agar plates and the bacterial colonies were counted. Data are expressed as means ± SD. **P < 0.05 compared to the PBS + sham group. *P < 0.05 compared to the PBS + CLP group. ^#^ *P* < 0.05 compared to the TsES + CLP group.

A systemic increase of proinflammatory cytokines is characteristic of the proinflammatory response and onset of sepsis. The serum concentration of cytokines was measured in mice 12 h after the CLP operation. Treatment with TsES significantly attenuated the elevated levels of TNF-*α*, IL-1*β*, IL-6, and HMGB1 after CLP. In addition, the regulatory factors IL-10 and TGF*β*1 were significantly elevated in the TsES + sham, PBS + CLP, and TsES + CLP groups compared to the PBS + sham group. Furthermore, the serum levels of IL-10 and TGF*β*1 were much higher in the TsES + CLP group than in the other groups (Figure 2(a)). The results indicate TsES may inhibit inflammatory response via increasing IL-10 and TGF*β*1.

Peritoneal white blood cells were also analyzed after the CLP operation. Mice in the TsES + CLP group had more peritoneal neutrophils and fewer monocytes 4 h and 12 h after the operation than the PBS + CLP group. The two groups were no longer different 24 h after the operation (Figure 2(b)). The peritoneal and blood CFU of bacteria were not different 4 h after operation regardless of treatment with TsES (Figures 2(c) and 2(d)). However, 24 h after the operation, TsES treatment had reduced the peritoneal and blood CFU of bacteria in mice after CLP (Figures 2(c) and 2(d)).

### 3.3. TsES Downregulated MyD88/NF-*κ*B Signaling but Had No Effect on TLR2 and TLR4 Expression in Peritoneal Macrophages from Septic Patients

**Figure 3:**
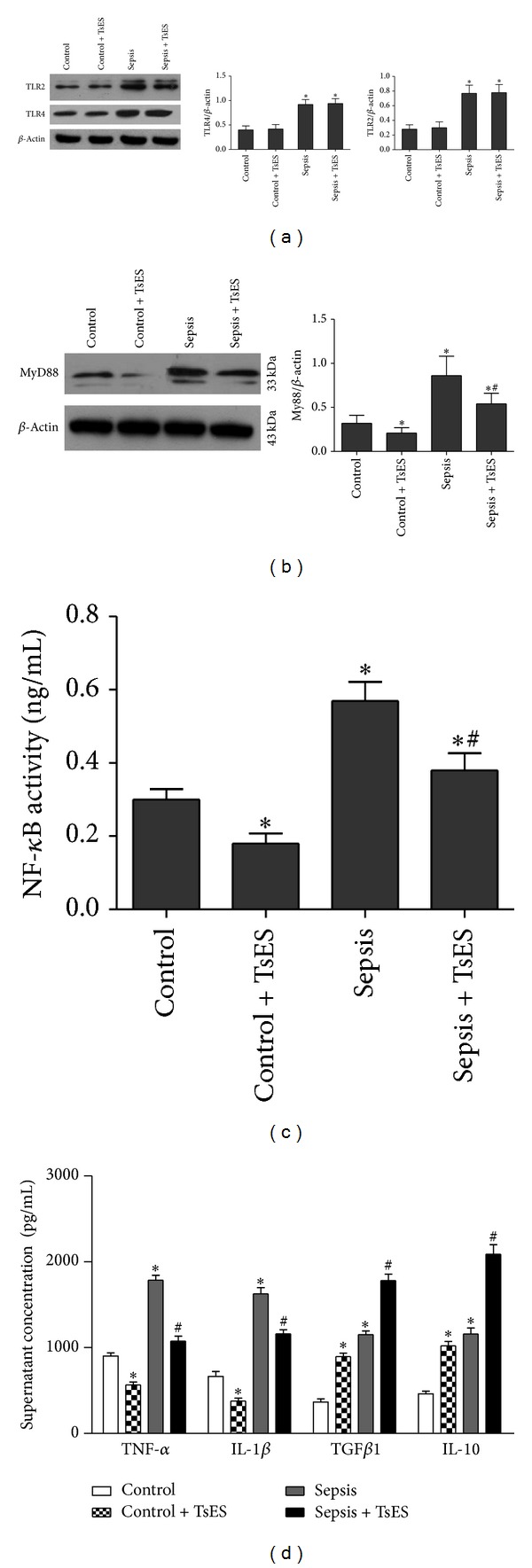
The effects of TsES on peritoneal macrophages in septic patients. Macrophages were collected from peritoneal lavage or abdominal cavity flushing fluid in 27 septic patients and 16 control patients and then treated with TsES (5 *μ*g/mL) or the same volume of PBS. Membrane protein fractions were extracted after 6 h in culture. The expressions of TLR2 and TLR4 were detected by western blot (a). The cytoplasmic proteins were extracted after 6 h in culture. MyD88 expression in macrophages was detected by western blot (b). NF-*κ*B activity in nuclear extractions was determined using the NF-*κ*B p50/p65 Transcription Factor Assay Kit (c). Culture supernatants were collected after 6 h in culture. Supernatant TNF-*α*, IL-1*β*, IL-10, and TGF*β*1 levels were detected by ELISA (d). Data are mean ± SD of three independent experiments, analyzed in triplicate. *P < 0.05 compared to the control group. ^#^ *P* < 0.05 compared to the sepsis group.

We enrolled 27 septic patients and 16 control patients without infection after abdominal surgery. The main diagnoses for the septic patients were liver cancer (*n* = 6, 22%), colon cancer (*n* = 7, 26%), spleen rupture (*n* = 4, 15%), cholecystitis (*n* = 6, 22%), and intestinal perforation (*n* = 4, 15%). The main diagnoses for the control patients were liver cancer (*n* = 4, 25%), colon cancer (*n* = 4, 25%), spleen rupture (*n* = 2, 13%), cholecystitis (*n* = 3, 19%), and intestinal perforation (*n* = 3, 19%). There were no significant differences in the general characteristics (e.g., age, sex, etc.) of the two groups. Macrophages were collected from the peritoneal lavage or abdominal cavity flushing fluid. We examined the effects of TsES on expression of TLR2 and TLR4 on macrophages after 6 h in culture. Macrophages from septic patients had higher cell surface expression of both TLR4 and TLR2 compared to the control group. Exposure to TsES did not affect TLR2 or TLR4 expression (Figure 3(a)). However, TsES downregulated the key TLR signal transducer MyD88 in macrophages from septic patients (Figure 3(b)). We also found that TsES inhibited the activity of NF-*κ*B in macrophages from septic patients (Figure 3(c)). After 6 h in culture, the supernatant was collected. TsES significantly reduced the supernatant levels of TNF-*α* and IL-1*β* produced by macrophages from septic patients and significantly increased the supernatant levels of IL-10 and TGF*β*1 (Figure 3(d)). In addition, TsES reduced MyD88 expression and NF-*κ*B activity, and regulated inflammatory cytokine production in macrophages from control patients (Figures 3(b), 3(c), and 3(d)).

### 3.4. TsES Upregulated MR Suppressing MyD88/NF-*κ*B Signaling* In Vitro*

**Figure 4:**
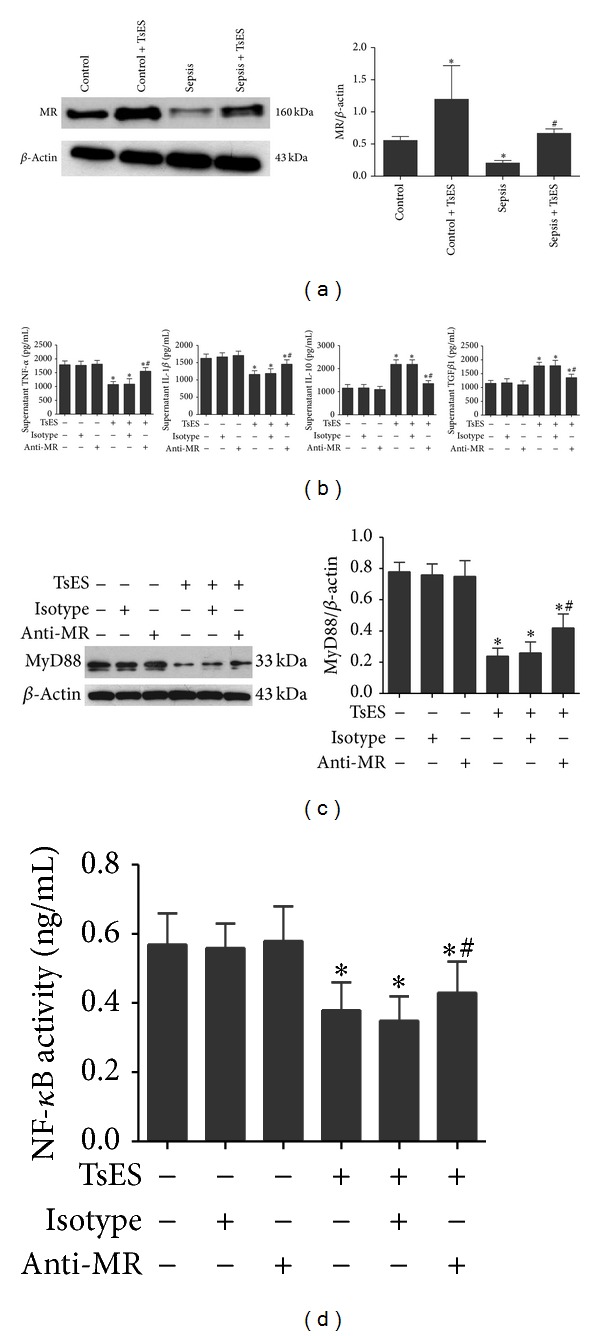
TsES suppressed MyD88/NF-*κ*B signaling via MR on peritoneal macrophages from septic patients. Macrophages from peritoneal lavage or abdominal cavity flushing fluid were collected from septic patients and the control group. Peritoneal macrophages were treated with TsES (5 *μ*g/mL) for 6 h. Membrane protein fractions were extracted. MR expression was detected by western blot. *P < 0.05 compared to the control group. ^#^ *P* < 0.05 compared to the sepsis group (a). Peritoneal macrophages from septic patients were treated with a specific blocking antibody to MR (5 *μ*g/mL) for 2 h and then with TsES for 6 h. Supernatant TNF-*α*, IL-1*β*, IL-10, and TGF*β*1 levels were detected by ELISA (b). MyD88 expression in macrophages was detected by western blot (c). NF-*κ*B activity was determined using the NF-*κ*B p50/p65 Transcription Factor Assay Kit (d). Data are mean ± SD of three independent experiments, analyzed in triplicate. *P < 0.05 with respect to macrophages in medium alone. ^#^ *P* < 0.05 with respect to macrophages stimulated with TsES.

We then determined the level of MR expression on macrophages from septic and control patients. Macrophages from septic patients had significantly lower MR expression compared to the control patients. TsES significantly increased MR expression on macrophages both from septic patients and from control patients after 6 h of incubation (Figure 4(a)). Next, to examine if the anti-inflammatory effects of TsES were mediated via MR, we preincubated macrophages from septic patients with a blocking antibody specific to MR (5 *μ*g/mL) for 2 h and then with TsES for 6 h. The MR blocking antibody partially reversed the TsES-mediated reduction in MyD88 expression in macrophages from septic patients, leading to increased MyD88 expression compared to TsES alone (Figure 4(c)). Similar results were obtained for NF-*κ*B activity and supernatant TNF-*α* and IL-1*β* in macrophages from septic patients preincubated with anti-MR and then stimulated with TsES (Figures 4(b) and 4(d)). In contrast, MR blocking partially inhibited the TsES-mediated increase in supernatant IL-10 and TGF*β*1 (Figure 4(b)). Therefore, it is likely that TsES suppressed MyD88/NF-*κ*B signaling via the MR on macrophages* in vitro*.

### 3.5. TsES Protected Mice from Sepsis via MR

**Figure 5:**
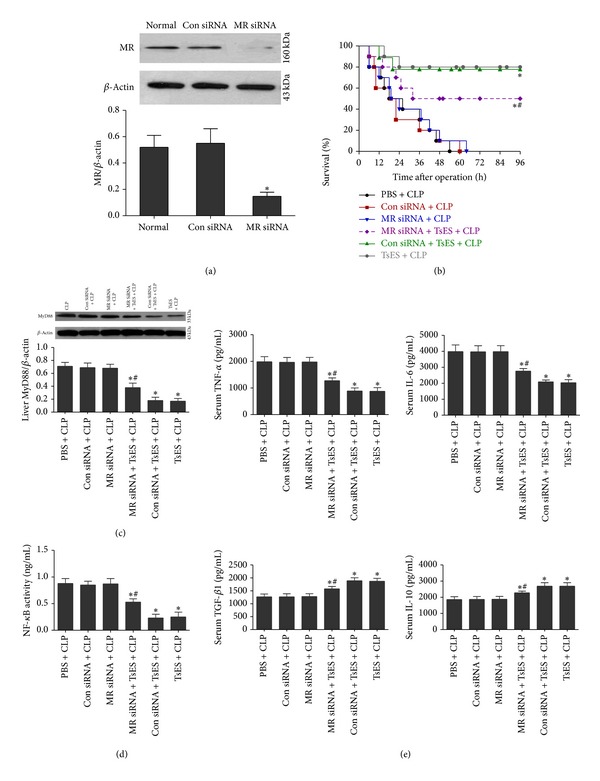
TsES protected mice from sepsis via MR. Mice were pretreated with MR siRNA and then underwent the CLP operation. On the day of the operation, MR expression on peritoneal cells was detected by western blot. *P < 0.05 compared to the normal group (a). The survival rate was assessed in six groups of mice (*n* = 10 mice/group): (1) PBS + CLP, (2) con siRNA + SLP, (3) MR siRNA + CLP, (4) MR siRNA + TsES + CLP, (5) con siRNA + TsES + CLP, and (6) TsES + CLP. The survival rate was analyzed 4 days after the CLP operation using the log-rank test (b). Twenty-four hours after CLP liver MyD88 expression (c), NF-*κ*B activity (d), and serum cytokine levels (TNF-*α*, IL-1*β*, IL-10, and TGF*β*1) (e) were determined. Triplicate independent experiments were analyzed. *P < 0.05 compared with the PBS + CLP group. ^#^ *P* < 0.05 compared to the TsES + CLP group.

Mice were injected via the tail vein with 5 *μ*g of MR siRNA or con siRNA 3 and 5 days prior to the CLP operation.  Figure 5(a) shows that MR siRNA inhibited the expression of MR on peritoneal cells in mice on the operation day. The survival rate was not changed after CLP in mice treated with only con siRNA or MR siRNA. As expected, TsES improved the survival rate of septic mice. However, the survival rate of mice treated with MR siRNA, but not con siRNA, and TsES was significantly lower than TsES alone (Figure 5(b)). The TsES-mediated decrease in liver MyD88 expression in septic mice was also partially reversed by MR siRNA at 24 h after the operation (Figure 5(c)). Similar results were obtained for liver NF-*κ*B activity and the serum concentrations of TNF-*α* and IL-1*β* from septic mice treated with MR siRNA and then with TsES (Figures 5(d) and 5(e)). Finally, the TsES-mediated increase in serum IL-10 and TGF*β*1 in septic mice was partially inhibited by MR siRNA (Figure 5(e)).

## 4. Discussion

The murine CLP-induced sepsis model was chosen for this study because the pathology associated with this model is similar to that observed during clinical peritonitis [19]. Systemic inflammation, organ damage, and high rate of mortality are characteristics of severe sepsis. This study demonstrated that treatment with TsES reduced mortality, CLP-induced liver, and renal damage and protected organ function in mice with CLP-induced polymicrobial sepsis. TsES also resulted in more peritoneal neutrophils and fewer monocytes in early CLP-induced sepsis and an increased ability to clear peritoneal and blood bacteria. These results suggest that TsES may enhance neutrophils recruitment leading to bacterial clearance and reduce local monocytes alleviating inflammatory response. Furthermore, administration of TsES to septic mice significantly reduced the serum levels of several inflammatory cytokines (TNF-*α*, IL-1*β*, IL-6, and HMGB1) and elevated regulatory factors (IL-10 and TGF*β*1). This shift indicates that TsES could exert its anti-inflammatory effects by inhibiting the inflammatory response. Some anti-inflammatory factors have been shown to activate immune cells to enhance bacterial clearance [22]. Previous studies demonstrate that anti-inflammatory agents such as monoclonal antibodies to TNF increase septic shock lethality due to suppression of the host's ability to fight infection [23]. TsES not only have anti-inflammatory effects but also enhance bacterial clearance. Interestingly, TsES have been very important in provoking host defense mechanisms against infections, both innate and adaptive [13]. Thus, TsES are a promising candidate for protecting against sepsis-induced mortality in patients.

TLR play an important role in the induction of the hyperinflammatory response and tissue injury in sepsis. TLR2 and TLR4 have been regarded as the main sensors for recognition of pathogen-associated molecular patterns from gram-positive and gram-negative bacteria, respectively [24]. Pathogens from abdominal infection are commonly mixed infection with gram-negative and gram-positive bacteria. Not surprisingly, TLR2 and TLR4 expression was increased in macrophages from septic patients. While TsES did not change the TLR2 and TLR4 expression, it dampened MyD88 in macrophages from septic patients. MyD88 is a key protein in the signaling pathway initiated by TLR, which can be of significant importance during sepsis [25]. MyD88^(−/−)^ mice subjected to sepsis had higher survival rate compared to the wild mice with sepsis [26]. Some agents that inhibit MyD88 expression can ameliorate murine polymicrobial sepsis [27]. TsES may improve the survival rate of septic mice by inhibiting MyD88. TsES were also able to dampen NF-*κ*B activity in macrophages from septic patients. By inhibiting both MyD88 and NF-*κ*B activity, TsES treatment may lead to fewer inflammatory factors during sepsis. There are currently 11 known mammalian TLR. Each TLR clearly has a unique role in generating the immune response [28]. Helminth parasites can activate or negatively regulate TLR to affect the immune response. For example,* Brugia malayi* downregulated mRNA expression of TLR3, TLR4, TLR5, and TLR7 on monocyte-derived DC [29]. The phosphorylcholine-containing glycoprotein ES-62 of the* filarial nematode* was also found to inhibit TLR4-MyD88, leading to the reduction of production of IL-12 and TNF-*α* [30]. While TsES did not affect expression of TLR2 and TLR4 during sepsis, it might still regulate other TLR expressions and should be further investigated.

Morelle et al. showed that glycans released from* T. spiralis* have a large number of mannose type structures [31]. These structures could be potential ligands that contribute to activation of the innate immune response of the host [32]. In the present study, TsES upregulated MR on peritoneal macrophages from septic patients. MR binds to microbial structures containing mannose to mediate endocytosis and phagocytosis that are part of the innate host defense against pathogen [33]. Thus, the TsES-mediated increase in the level of MR could contribute to enhanced bacterial clearance during sepsis. CLR triggering by different pathogens can induce diverse immune responses. The underlying signaling processes are complex and depend on crosstalk with other PRRs, the ligand or carbohydrate specific signaling pathway [34]. Studies have shown that there is crosstalk between TLR and CLR. Another member of the CLR family, DC-SIGN, modulates TLR signaling at the level of the transcription factor NF-*κ*B [35]. In this study, TsES reduced MyD88 and NF-*κ*B expression during sepsis. After blocking MR, the ability of TsES to inhibit MyD88 and NF-*κ*B expression in peritoneal macrophages from septic patients was attenuated. Furthermore,* in vivo*, blocking signaling through the MR using RNAi reduced the survival rate in CLP-induced septic mice treated with TsES. The anti-inflammatory effects of TsES, including inhibiting MyD88 and NF-*κ*B, and TNF-*α* and IL-1*β* were also reduced in CLP-induced mice after MR RNAi injection. These results show that TsES protected mice from polymicrobial sepsis via MR activation, which could engage in crosstalk with TLR signaling to inhibit MyD88 and NF-*κ*B. Ligand binding to MR may have an immunosuppressive function characterized by a profile of anti-inflammatory cytokines, which could prevent the generation of Th1-polarized responses [36], although, in other infection models like* Candida albicans*, MR can lead to increase inflammatory cytokines production and Th17 immune responses [37, 38]. Other* in vivo* studies have also shown that the engagement of MRs on macrophages by parasite ES products downregulates expression of IL-12 p40 and may compromise priming of the immune response [39]. TsES also have a regulatory function and increase IL-10 and TGF*β*1, which could regulate the inflammatory response during sepsis. MR is also a marker of alternatively activated macrophages (AAM). TsES-mediated enhanced MR expression in macrophages from septic patients could indicate increased AAM, another possible mechanism for TsES to exert its regulatory effects.

In conclusion, TsES improved survival in a mouse model of CLP-induced polymicrobial sepsis via an MR-dependent pathway. The survival benefit was associated with accelerated bacterial clearance and reduced proinflammatory response. In the context of sepsis, research efforts have been directed toward finding a mechanism to inhibit MyD88 and NF-*κ*B and limit the excessive inflammation triggered by TLR. Given its effects on MyD88 and NF-*κ*B, TsES might be a suitable novel therapeutic tool for treating sepsis.
